# On the interface between cultural transmission, phenotypic diversity, demography and the conservation of migratory ungulates

**DOI:** 10.1098/rstb.2024.0131

**Published:** 2025-05-01

**Authors:** Brett Jesmer, Janey Fugate, Matthew Kauffman

**Affiliations:** ^1^ Department of Fish and Wildlife Conservation, Virginia Tech, Blacksburg, VA 24061, USA; ^2^ Wyoming Cooperative Fish and Wildlife Research Unit, Department of Zoology and Physiology, University of Wyoming, Laramie, WY 82071, USA; ^3^ United States Geological Survey, Wyoming Cooperative Fish and Wildlife Research Unit, University of Wyoming, Laramie, WY 82071, USA

**Keywords:** animal culture, cultural evolution, ideal free distribution, natal habitat preference induction, niche variation hypothesis, portfolio effect, site fidelity, spatial personalities

## Abstract

Recent evidence indicates that green-wave surfing behaviour in ungulates and the migrations that stem from this behaviour are socially learned, culturally transmitted across generations and become more efficient via cumulative cultural evolution. But given a lack of corroborative evidence, whether ungulate migration is a cultural phenomenon remains a hypothesis deserving of further testing. In this opinion piece, we summarize the role memory and social learning play in the green-wave surfing that underlies ungulate migration, and when combined with the natural history of ungulates, we argue that the most likely mechanism for maintenance of ungulate migration is animal culture. We further our argument by providing a synopsis of processes that promote diversification of migratory behaviour and link these processes to their emergent ecological patterns, which are common in nature but have not historically been considered as potential cultural phenomena. The notion that diverse portfolios of migratory behaviour may buffer populations from environmental change emerges from this synthesis but requires empirical testing. Finally, we contend that, because the migratory behaviour of ungulates stems largely from cultural transmission as opposed to a genetic programme, the diversity of observed migratory strategies represents ‘culturally significant units’ deserving of the same conservation effort afforded to evolutionarily significant units.

This article is part of the theme issue ‘Animal culture: conservation in a changing world’.

## 1. Introduction

Ungulates—hooved mammals belonging to orders Cetartiodactyla and Perissodactyla—often make long, complex seasonal movements that are choreographed to match pulses in resources across space and time [1]. Ungulates commonly select forage at an intermediate phenological state wherein plant biomass and digestibility are optimized to maximize energy and nutritional gain per unit effort [2–4]. When gradients of plant phenology progress across landscapes in predictable ways, ‘green waves’ of high-quality forage emerge [5]. Migratory ungulates track, or ‘surf’, these waves across the landscape—a behaviour known as ‘green-wave surfing’ [6,7]—often resulting in increased fitness via greater exposure to, and intake of, energy and nutrients [8–10]. Ungulate migration can therefore be viewed as a behavioural adaptation to landscapes where predictable spatiotemporal resource gradients exist, an adaptation that has emerged many times throughout the history and phylogeny of ungulates [11].

Although conserving migratory behaviour is critical for sustaining ungulate populations, understanding how migratory behaviour develops and is maintained remains an enduring challenge for ecologists, thereby limiting the information available to policymakers interested in conserving migratory species [1,12]. Nevertheless, recent empirical evidence and traditional ecological knowledge suggest that the migratory behaviour of ungulates, their migration routes and their selection of seasonal ranges are not encoded primarily in their genome but are rather group-typical patterns of behaviour born from socially learned and transmitted information [13,14]—a phenomenon referred to as animal culture [15,16] (see table 1). As detailed below, researchers have demonstrated that green-wave surfing behaviour and the migrations that stem from such surfing (i.e. movements) are socially learned via horizontal (i.e. within a generation), vertical (i.e. parent–offspring) or oblique (i.e. non-parental conspecific from a different generation) cultural transmission, are further refined via asocial learning, and become more efficient over time via a process known as cumulative cultural evolution ([13,20,21]; see table 1 for details). However, empirical evidence supporting a cultural basis for ungulate migration is only beginning to emerge, and the mechanisms underlying the diversification and adaptation of migratory strategies are not well understood [39].

**Table 1:** Glossary.

term	definition	citation(s)
social learning	learning facilitated by an observer interacting with a model (another individual or its products)	[15,17,18]
asocial learning	learning facilitated by an individual interacting with its environment and through trial and error rather than learning from a model	[17,18]
cultural transmission	information, knowledge or behaviour(s) transmitted through a group of individuals via social learning	[19]
cumulative cultural evolution	accumulation of knowledge through repeated cycles of asocial learning (e.g. innovation) and social learning such that the knowledge base is improved upon within and across generations (i.e. via horizontal, vertical and oblique transmission; also referred to as the ‘ratcheting effect’)	[20–22]
Darwinian cultural evolution	process by which Darwinian selection pressures influence the frequency and geographical distribution of culturally transmitted behaviours	[23–25]
ideal free distribution hypothesis	individuals distribute themselves in a density-dependent manner across habitat types in a way that maximizes their fitness	[26,27]
niche variation hypothesis	groups of individuals specialize on different subsets of biotic or abiotic conditions (i.e. intra-specific niche partitioning) in a way that maximizes their fitness and facilitates greater population densities	[28–31]
natal habitat preference induction hypothesis	experience with natal habitat conditions shapes the post-dispersal habitat preferences of individuals and is a source of phenotypic variation in habitat selection	[32]
spatial personality hypothesis	consistent individual differences in spatial behaviour (e.g. movement strategies, habitat use and selection)	[33–35]
culturally significant units	particular variants of a cultural trait (e.g. migratory versus resident behavioural strategies, or different migratory routes used to connect seasonal ranges)	[36]
portfolio effect hypothesis	intra-specific trait variation buffers population-level demographic rates against environmental variability via selection for and against specific traits over time and space	[37,38]

**Figure 1.**
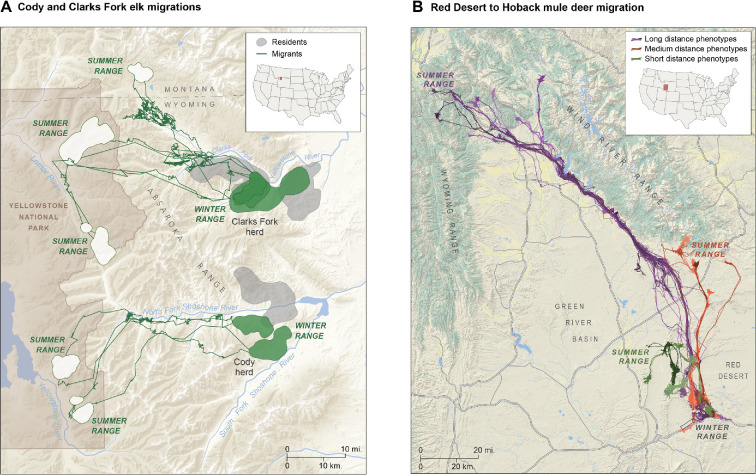
Maps depicting phenotypic diversity in movement and space-use strategies. (A) Illustration of partially migratory elk (*Cervus canadensis*) herds in the Greater Yellowstone Ecosystem, USA. A sample of five migratory (green and white shaded areas and green lines) and three resident (grey areas) elk in the Clarks Fork and Cody, Wyoming, herds provide an example of the phenotypic diversity present in these populations. (B) Illustration of phenotypic diversity within an obligate migratory herd of mule deer (*Odocoileus hemionus*). All mule deer reside together in the Red Desert of Wyoming, USA, during winter but exhibit three different migratory strategies and summer range destinations. During spring, long-distance migrants (purple) leave the Red Desert and navigate their way to the Hoback Basin of the Wyoming Range, where they spend the summer. Medium-distance migrants (orange) summer in the southern Wind River Range. Short-distance migrants (green) summer on ridge tops within the Red Desert. Maps adapted from *Wild migrations: atlas of Wyoming’s ungulates* [44].

**Figure 2.**
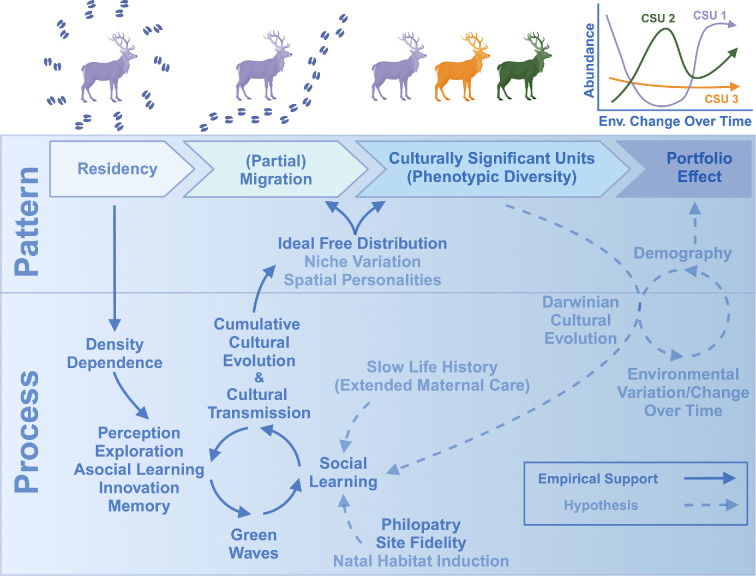
Hypothesized and empirically supported pathways by which ungulate migrations (i) are developed (i.e. transition from resident to migratory), (ii) are maintained via social learning and cultural transmission, (iii) become more efficient through cumulative cultural evolution, (iv) diversify into distinct culturally significant units, (v) adapt to environmental change through Darwinian cultural evolution, and (vi) facilitate population persistence via the portfolio effect. CSU, culturally significant unit. Created using https://BioRender.com.

Efforts to map ungulate migrations across western North America and elsewhere across the globe have highlighted a great diversity of migratory strategies within ungulate populations ([40–43]; e.g. figure 1). Several longstanding ecological concepts, such as the ideal free distribution [26], the niche variation hypothesis [28] and the portfolio effect [37,45] (see table 1), provide conceptual frameworks upon which ecologists can build and advance knowledge regarding how migratory behaviours diversify and adapt to environmental change (figure 2). Yet, tests of the linkages among these ecological concepts and animal culture are rare (for examples of diversification and adaptation of other ungulate foraging and movement behaviours, see [46–48]). By understanding the behavioural and environmental mechanisms underlying diversification of migratory portfolios and their adaptive benefits, policymakers would be better able to make informed decisions regarding land and population management.

**Figure 3.**
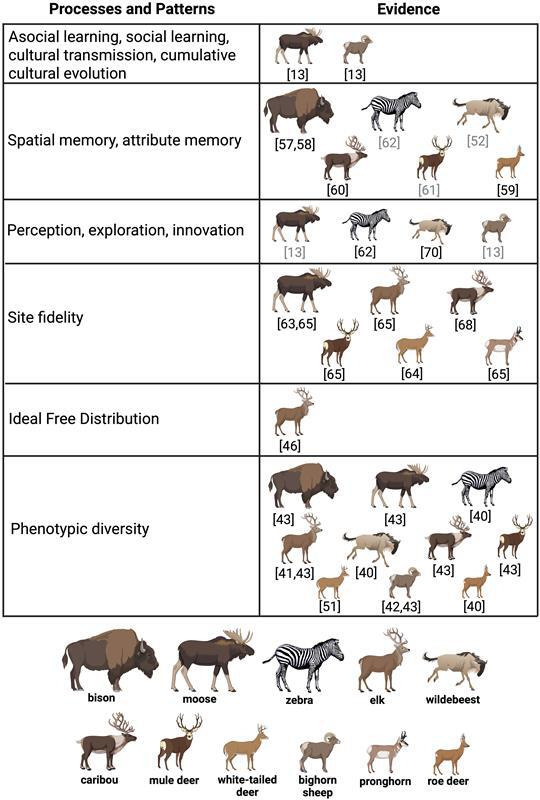
Summary of direct evidence (citations in black; i.e. empirical evidence) and indirect evidence (citations in grey; e.g. evidence derived from simulation studies, author conclusions, or logical deduction) of the processes and patterns described in the text and illustrated in figure 2. Created using https://BioRender.com.

Given the challenges of demonstrating social learning and animal culture in free-ranging wildlife [49], we followed the framework provided by Brakes *et al*. [36] for demonstrating potential for social learning and animal culture. Specifically, we sought to (i) summarize literature directly indicating, and indirectly suggesting, a cultural basis for ungulate migration (e.g. spatial and attribute memory, social learning and cultural transmission; for a comprehensive review, see [50]); (ii) discuss the cultural processes that promote diversification of ungulate movement and foraging behaviour and link these processes to their emergent ecological patterns; and (iii) summarize theoretical and empirical evidence supporting the notion that diversification of migratory strategies increases the capacity of ungulates to adapt to environmental change (figure 3). We finish by providing a brief overview of the concept of conserving evolutionarily (i.e. genetic) significant units to safeguard biodiversity and discuss how extending this concept to include ‘culturally significant units’ may help safeguard the diverse repositories of cultural knowledge [36] possessed by ungulates living on seasonal landscapes and promote healthy, abundant populations.

## 2. Evidence that social learning, culture and cultural evolution underlie ungulate migrations

Ecologists have long hypothesized that the migratory behaviour of ungulates stems primarily from social learning and cultural transmission of the spatial knowledge needed to effectively track resources (i.e. green waves) and navigate between seasonal ranges [51–53]. To date, the best available evidence of social learning of migratory behaviour in ungulates comes from a series of translocations wherein migratory bighorn sheep (*Ovis canadensis*) and moose (*Alces americanus*) were moved into landscapes where green waves existed and migratory behaviour would have been an adaptive strategy [13]. But translocated individuals had no prior knowledge of the spatiotemporal patterns of green waves and did not initially exhibit migratory behaviour, indicating that migratory behaviour is not solely based on the ability of ungulates to perceive green waves nor is it under strong genetic control. After decades of opportunity to learn about green waves both socially and asocially, however, the ability of animals in this study to track green waves across the landscape increased, resulting in the emergence of migratory behaviour. Further, when individuals were translocated into populations with knowledge of local green waves and where some individuals had already begun to express migratory behaviour, translocated individuals began migrating in just 1−2 years, thus indicating horizontal transmission of information (i.e. social learning; [13]). The most plausible explanation for the observed increase in knowledge regarding spatiotemporal patterns of green waves over decades is that information was culturally transmitted horizontally, vertically or obliquely across generations and improved upon by each generation via asocial learning—a process known as cumulative cultural evolution ([13,20,22]; see table 1).

Learning requires information to be stored in memory, upon which animals can base their decision-making [54]. With regard to ungulate foraging and migratory behaviour, the ability to learn socially or asocially about green waves requires two specific types of memory: spatial memory (i.e. encoding of spatial relationships [55]) and attribute memory (i.e. encoding of locale-specific characteristics [53,56]). Indeed, the role of spatial and attribute memory in ungulate foraging ecology has become well established in the literature over the past decade. For example, bison (*Bison bison*), roe deer (*Capreolus capreolus*) and caribou (*Rangifer tarandus*) encode information about the location and quality of resource patches into memory and use this information to optimize their patch-to-patch movements in a way that maximizes energy and nutrient intake [57–60]. Further, the seasonal range and migration route selection of mule deer (*Odocoileus hemionus*), zebra (*Equus burchelli*) and wildebeest (*Connochaetes taurinus*) could not be recreated using simulated animal movements that relied on perception alone. Only when simulated movements were parameterized with memory of migratory routes and seasonal ranges could observed migratory routes and seasonal ranges be recreated [52,61,62]. Thus, evidence that ungulates rely on memory to guide their movement and foraging decisions suggests that social learning and cultural transmission likely play a key role in the development and maintenance of their migratory behaviour (figure 2).

Ungulates tend to return to or maintain distinct seasonal ranges and migratory paths year after year—a phenomenon known as site fidelity [63–65]. Because it is only beneficial to exhibit such site fidelity if high-quality resources are present at a given time and place [66], the degree to which individuals should exhibit such site fidelity depends on the spatiotemporal predictability of resources [65,67]. Spatial and attribute memory underlies the ability of individuals to act on information regarding the spatiotemporal predictability of resources and hence continuously return to profitable areas (e.g. [57]). For example, 60 years of migratory caribou data indicate that adult females chose calving areas that did not necessarily possess optimal foraging conditions in a given year but rather that over decades had predictably above-average forage conditions compared with the landscape as a whole [68]. The most plausible explanation for this result is that caribou had encoded information about the forage attributes of specific locations on the landscape (i.e. leveraged attribute memory) and used spatial memory to navigate back to historic calving grounds [68]. For the calving ground locations to remain unchanged for at least six decades, calving ground information was most likely transmitted vertically or obliquely across generations via social learning.

Information about migratory routes and seasonal ranges can also be transmitted indirectly in the form of perceptual cues (i.e. visual or olfactory signals) left behind by other animals during past or present migrations—a type of social learning known as ‘local enhancement’ [67,69]. For instance, migration paths beaten into the ground over generations are thought to contain social information in the form of a visual cue useful to ungulates learning to navigate between seasonal ranges [70]. And volatile organic compounds (i.e. scent) left behind by inter-digital glands on the hooves of ungulates are thought to chemically communicate information about migration routes and seasonal ranges in wildebeest [70]. Natal habitat preference induction also stems from social learning, is relevant to the maintenance of migratory behaviour and may involve local enhancement [32]. The natal habitat preference induction hypothesis states that, because natal habitat serves as a cue that natal habitat conditions can support survival and reproduction, offspring are expected to develop habitat and space-use preferences similar to those experienced in their natal range ([32,71]; see table 1). In the context of migration, the natal habitat preference induction hypothesis suggests that offspring should adopt similar movement behaviour (i.e. migratory or resident) to that of their mothers (e.g. [72,73]). Visual, olfactory and natal habitat cues therefore provide information that can be stored in memory and transmitted among individuals, thereby representing a form of social learning. Migratory traditions may therefore be continually reinforced by the persistence of such cues, further cementing migratory culture into populations. Given the role of spatial memory in shaping ungulate movements, as well as the long-established role of local enhancement across the animal kingdom, growing evidence indicates that ungulates use socially transmitted information about the location of resource patches, migration corridors, and seasonal ranges in their decision-making (figure 2).

When the demonstrated importance of spatial memory, attribute memory, social learning, asocial learning and natural history characteristics of ungulates (i.e. site fidelity, prolonged maternal care, philopatry of females, chemical and visual communication) are combined, a case can be made that migratory and foraging behaviour in many ungulate taxa is socially learned, culturally transmitted and thus subject to cultural evolution (figure 2). In contrast to classical Darwinian (i.e. genetic) evolution, the learning mechanisms underlying cultural evolution provide animal populations with the capacity to rapidly adapt to environmental change occurring at ecological rather than evolutionary timescales [23]. There are two pathways by which cultural evolution may occur: (i) revolution and wholesale behavioural change at the population level [74,75], or (ii) culturally maintained phenotypic diversification and selection for or against certain phenotypes (i.e. Darwinian cultural evolution, table 1; [23,36]). Understanding if and how cultural evolution may buffer migratory ungulates against environmental change therefore represents an important and understudied aspect of ungulate conservation (for example of culture resulting in maladaptive behaviour, see [76]).

## 3. The interface between cultural transmission, intra- and inter-population phenotypic diversity, fitness and the conservation of migratory ungulates

The concept of conserving evolutionarily significant units has been in practice for at least 40 years [77]. Because evolutionarily significant units are genotypically distinct groups of animals, they may possess greater phenotypic diversity (i.e. among-individual variation in behavioural, physiological or morphological traits), more readily adapt to environmental change and hence facilitate population persistence amid environmental change [78,79]. For this reason, several authors have suggested that conservation outcomes would be improved if both genotypic and phenotypic diversity were considered when delineating evolutionarily significant units (e.g. [80–82]). Likewise, phenotypic variation in behaviour stemming from social learning and cultural transmission (hereafter, ‘culturally significant units’; also known as ‘cultural variants’, table 1; [36]) warrants consideration. For example, many populations of cetaceans (i.e. whales, dolphins and porpoises) comprise culturally significant units reflecting socially learned foraging, migratory and song traditions that influence resource acquisition in response to variation in environmental conditions [83–85]. For this reason, conservation policy has aimed to protect culturally significant units of cetaceans [36]. Nevertheless, and despite evidence that harnessing the phenotypic diversity exhibited among culturally significant units can improve conservation outcomes, the diversity of animal cultures has been historically undervalued by conservationists and policymakers working to conserve ungulate populations [12,36].

Populations of migratory ungulates exhibit phenotypic variation in their movement behaviour along a gradient from resident to migratory behaviour. While many ungulate populations exhibit obligate resident or migrant behaviour (e.g. [6,73,86]), it is also common for populations to exhibit partially migratory behaviour wherein part of the population migrates and part of the population does not ([87–89]; figure 1). In partially migratory populations, some individuals may switch between migratory and resident strategies using win–stay–lose–switch decision-making [65]—a notion referred to as facultative migration [46]. The ability of individuals to employ the win–stay–lose–switch strategy, which allows them to switch between two or more alternative seasonal ranges or migratory routes, stems from perception, memory and likely social learning. For this reason, among-individual variation in migratory strategy exists within both obligate and partially migratory populations. For instance, groups of individuals within populations of mule deer, bighorn sheep and elk (*Cervus canadensis*) use different migration corridors that end at either shared or distinct seasonal ranges ([41–43,88,90,91]; figure 1), and these phenotypes exhibit equivocal or disparate population growth rates (i.e. fitness), suggesting these phenotypes are subject to selective pressures stemming from spatiotemporal variation in environmental conditions. Thus, if inter- and intra-population diversity in migratory strategies stem from cultural transmission, each strategy may represent a culturally significant unit deserving of conservation attention similar to how conservation efforts have benefitted from a focus on genetically significant units over the past four decades [36,78,80,81].

Three non-mutually exclusive hypotheses may help explain the development and maintenance of phenotypic diversity in migratory strategies: the notions of the ideal free distribution hypothesis, the niche variation hypothesis and the spatial personalities hypothesis (figure 2 and table 1). In the context of migration, the ideal free distribution hypothesis states that at high-population densities some individuals will develop movement tactics to exploit lower-quality habitat to reduce competition for resources. By individuals distributing themselves in a density-dependent manner, among-group fitness should equalize and be maintained at a higher level than if all groups continued to compete for the same set of finite resources [26,92]. This is conceptually similar to the niche variation hypothesis [28], wherein individuals specialize in different subsets of environmental space and resources via fidelity to different routes or seasonal ranges and thereby increase population-level fitness by reducing intra-specific competition [29,30,93–95]. And the spatial personalities hypothesis, which is an extension of the animal personalities hypotheses (for review, see [96,97]) and is conceptually similar to the niche variation hypothesis, proposes that among-individual variation in migratory and space-use behaviour across time is greater than within-individual variation ([33]; table 1). Consistent with these hypotheses, more than two decades of research on the movement ecology and demography of elk in the Banff National Park region of Alberta, Canada, has demonstrated substantial among-individual variation in migratory and space-use behaviour, and that fitness is equalized between these different behavioural tactics (i.e. migration versus residency). Whereas migratory elk were in better nutritional condition and had higher reproductive rates than resident elk, resident elk had higher adult survival because a ‘human shield’ reduced predation by wolves (*Canis lupus*). Because these two strategies were demographically balanced, these strategies represent alternative evolutionarily stable states [46,98,99]. Evidence therefore suggests that the processes of social learning, asocial learning, cultural transmission and cultural evolution of knowledge regarding spatiotemporal distributions of resources may underlie emergent patterns consistent with the niche variation, spatial personalities and the ideal free distribution hypotheses (figure 2). Nevertheless, the conservation importance of if and how a diversity of movement strategies and alternative stable states may buffer ungulate populations against environmental change in the Anthropocene is not well understood.

The portfolio effect is a concept wherein intra-specific trait variation buffers population-level demographic rates against environmental variability via selection for and against specific phenotypes over time and space ([37,38]; figure 2 and table 1). Although the portfolio effect has not been directly demonstrated in ungulates, evaluations of fitness components across different migratory phenotypes suggest that migratory ungulate populations may benefit from the portfolio effect. For example, although average fitness over a 20 year period was not different across migratory phenotypes (i.e. two migratory and one resident strategy) of elk in the Banff National Park area, among-phenotype fitness varied over time, resulting in two phenotypes exhibiting higher abundance than the third during the first 10 years, yet the third phenotype exhibited greater than or equal abundance to the first two phenotypes in the second 10 year period [46]. Likewise, a partially migratory herd of elk in northwest Wyoming, USA, exhibited shifts in the relative fitness and abundance of long- versus short-distance migrants over 35 years. Because of changes in spatial distribution of human harvest and large carnivore abundance as well as human development near their winter range, the short-distance phenotype benefitted from a human shield, whereas the long-distance phenotype experienced reductions in survival, reproduction and abundance [100]. In the Red Desert and Sierra Nevada mountains of the USA, long-term monitoring (9 and 13 years, respectively) of migratory mule deer populations has demonstrated that some phenotypes have higher fitness than others. In both examples, populations of mule deer shared a common winter range but either migrated to different areas of their respective mountain ranges or were year-round residents on the winter range. In each case, one migratory phenotype exhibited superior fitness (as proxied by nutritional condition, reproductive rates and survival rates) relative to the other phenotype(s) [90,91]. Such among-phenotype variation in fitness may therefore provide the raw material needed for adaptation via the portfolio effect and Darwinian cultural evolution [23].

## 4. Conclusions

Direct evidence of social learning and cultural transmission of migratory behaviour in ungulates is only available for a few species inhabiting temperate environments, such as white-tailed deer (*Odocoileus virginianus*), mule deer, moose and bighorn sheep ([13,51,64,72,73]; figure 3). Yet when empirical evidence of social learning and cultural transmission is combined with the demonstrated importance of attribute and spatial memory in ungulate foraging behaviour, natural history traits (e.g. extended maternal care, philopatry), site fidelity and natal habitat induction, as well as indirect evidence from simulation studies (figure 3), an argument can be made that specific migration routes and seasonal ranges are maintained by cultural transmission across an array of ungulate species and systems ([101]; figure 2). Further, because social learning and cultural transmission are mechanistic processes by which patterns of phenotypic diversity consistent with predictions of the ideal free distribution hypothesis, niche variation hypothesis, spatial personality hypothesis and the portfolio effect emerge (figure 2 and table 1), and because the hypotheses are well supported in ungulates and other taxa, we expect the hypothesis that ungulate migration is a cultural phenomenon will continue to garner empirical support. Moreover, because movement is a key behaviour by which ungulates exploit their environment, we expect ecologists to continue to discover new ways in which social learning and culture underpin their space use.

Despite evidence suggesting the portfolio effect may be operating in migratory ungulate populations, empirical evidence is scant and important questions remain, highlighting the need for long-term data on migratory phenotypes and their demography. Without further investigation, the studies of migratory mule deer discussed in the above paragraph would suggest maladaptive phenotypes exist. To understand why seemingly maladaptive phenotypes exist requires considering the temporally limited scope at which contemporary research is typically conducted. To exist today, maladaptive phenotypes must have been adaptive under past environmental conditions or must be maintained by positive fitness outcomes under contemporary conditions occurring at frequent enough intervals to prevent phenotype loss [23,76,102]. Might a seemingly maladaptive resident strategy do better in big snowpack years? Might migrants fare better in average or drought years, but not in big snowpack years? Because most studies discussed here rely on measures of average fitness (e.g. average reproductive rate, survival rate or lambda) over a several-year period to make inference, we cannot yet fully ascertain whether ungulate populations are adapting to environmental change via Darwinian cultural evolution or if the portfolio effect is operating.

To understand if and how diverse portfolios of culturally significant units may buffer populations against environmental change through Darwinian cultural evolution, long-term studies of both the movement and demography of populations must be conducted. Scientists can use a variety of methods (e.g. biologging, acoustic monitors, stable isotopes, mark–recapture, etc.; for review, see Whiten & Rutz, this issue [49]) to not only track the movements of parental generations (P), but also track the movements, survival and reproduction of f_1_, f_2_, … f_n_ generations. By doing so, data capable of testing whether various movement and foraging behaviours are socially learned and culturally transmitted across generations would be generated [39] and could also be leveraged to identify culturally significant units and test if environmental variation leads to disparate demographic responses among culturally significant units (i.e. Darwinian cultural evolution and the portfolio effect; figure 2). Collecting such multigenerational data presents many logistical and financial challenges but could advance basic knowledge and improve conservation outcomes.

The exact ways in which climate change will alter spatiotemporal patterns of plant phenology at scales relevant to ungulate migration are uncertain, but models predict both directional shifts and increased inter-annual variability in temperature and precipitation, which will influence spatiotemporal patterns of plant phenology and the ability of ungulates to track green waves [103,104]. Hence, the social learning and cultural transmission that underlie culturally significant units may allow populations to adapt to environmental change much more rapidly than via classical Darwinian (i.e. genetic) adaptation [23,37]. For these reasons, and when the movement strategies of ungulates are either demonstrated to be or expected to be inherited via social learning and cultural transmission [13,51,64,72,73], efforts to facilitate phenotypic diversity and conserve culturally significant units in the face of unprecedented rates of climate and land-use change could promote long-term stability of ungulate populations. Culturally significant units, whether currently adaptive or maladaptive, may therefore provide a diverse portfolio of behaviours capable of buffering populations against environmental change.

## Data Availability

This article has no additional data.
